# Organ- and cell-specific immune responses are associated with the outcomes of intracerebral hemorrhage

**DOI:** 10.1096/fj.201700324R

**Published:** 2017-09-06

**Authors:** Jing Zhang, Kaibin Shi, Zhiguo Li, Minshu Li, Yujuan Han, Lei Wang, Zhecheng Zhang, Changlu Yu, Fang Zhang, Lijuan Song, Jing-Fei Dong, Antonio La Cava, Kevin N. Sheth, Fu-Dong Shi

**Affiliations:** * Department of Neurology, Tianjin Neurological Institute, Tianjin Medical University General Hospital, Tianjin, China;; † Department of Neurology, Tianjin Third Central Hospital, Tianjin, China;; ‡ Department of Radiology, Tianjin Third Central Hospital, Tianjin, China;; § Department of Neurology, Barrow Neurological Institute, St. Joseph’s Hospital and Medical Center, Phoenix, Arizona, USA;; ¶ 2011 Collaborative Innovation Center/Neurobiology Research Center, Shanxi University of Traditional Chinese Medicine, Shanxi, China;; ‖ Division of Hematology, Department of Medicine, Bloodworks Research Institute, University of Washington School of Medicine, Seattle, Washington, USA;; # Department of Medicine, David Geffen School of Medicine, University of California, Los Angeles, Los Angeles, California, USA;; ** Department of Neurology, Yale University School of Medicine, New Haven, Connecticut, USA

**Keywords:** hemorrhagic brain injury, immunosuppression, spleen, infection

## Abstract

Severe brain injury significantly influences immune responses; however, the levels at which this influence occurs and which neurogenic pathways are involved are not well defined. Here, we used MRI to measure spleen volume and tissue diffusion changes in patients with intracerebral hemorrhage (ICH). We observed increased capillary exchange and spleen shrinkage by d 3 post-ICH, with recovery by d 14. The extent of spleen shrinkage was associated with brain hematoma size, and a reduced progression of perihematomal edema was observed in the presence of severe spleen shrinkage. At the cellular level, lymphopenia was present in patients with ICH at admission and persisted up to 14 d. Lymphopenia did not parallel the observed spleen alteration. In addition, patients with ICH with infection had significant deficiencies of T and NK cells and poor functional outcomes. Finally, in mouse models of ICH, spleen shrinkage could be related to innervations from adrenergic input and the hypothalamus-pituitary-adrenal (HPA) axis. In sum, the profound impact of ICH on the immune system involves the coordinated actions of sympathetic innervation and the HPA axis, which modulate spleen shrinkage and cellular immunity.—Zhang, J., Shi, K., Li, Z., Li, M., Han, Y., Wang, L., Zhang, Z., Yu, C., Zhang, F., Song, L., Dong, J.-F., La Cava, A., Sheth, K. N., Shi, F.-D. Organ- and cell-specific immune responses are associated with the outcomes of intracerebral hemorrhage.

Intracerebral hemorrhage (ICH) accounts for debilitating brain damage in 10–15% of all patients who have suffered a stroke and carries a high rate of both morbidity and mortality (1). Current treatments for ICH are the surgical decompression of large, life-threatening hematomas and support therapies (2, 3). At the tissue level, the hematoma that follows ICH disrupts the brain’s cell architecture and promotes local inflammation that is characterized by the activation of microglia (4) and infiltration of peripheral immune cells (5–7) that accentuate brain injury (2, 8) and contribute to the formation of perihematomal edema (PHE), which amplifies local cell death. PHE increases in volume by approximately 75% in the first 24 h after ICH, peaks at approximately 5–6 d later, and lasts up to 14 d (1). The extent of PHE is considered an important predictor of ICH outcomes, and the attenuation of PHE by an immune modulator, fingolimod, in a pilot study improved clinical outcomes in patients with ICH and reduced plasma levels of matrix metalloproteinase-9 (9, 10).

The discovery of lymphopenia after ICH both in experimental models and in patients also suggests that ICH could impair peripheral immunity (11), which may be associated with increased infection complications (11–13). Infection is an important clinical complication after stroke. A meta-analysis of poststroke infection in a mixed group of 137,817 patients with mostly ischemic but also hemorrhagic stroke found a 30% overall infection rate, with pneumonia and urinary tract infection rates of 10% each (14). The reported incidence of infections after ICH was 11–58% (15–18), and, recently, a survey of 800 patients from a multicenter, triethnic population reported a 31% infection rate post-ICH (19). A retrospective study of 24,540 visits reported that infections were associated with a majority of 30-d readmissions after ICH as well as with increased mortality (20). Of interest, in patients with ICH, an autonomic shift detected by decreased baroreflex sensitivity was found to be associated with in increased susceptibility to infection (15); however, there is still limited knowledge of the effects of ICH on the immune system and related mechanisms as well as clinical association. Here, we investigated the organ- and cell-specific immune responses in relation to neurogenic pathways and clinical outcomes in ICH.

## MATERIALS AND METHODS

### Patients with ICH

Patients with ICH with the following criteria were recruited for this study: men and nonpregnant women age ≥ 18 yr with a primary supratentorial ICH of 5–40 cc (confirmed by computed tomography), symptom onset at <24 h before hospital admission, a Glasgow coma scale (GCS) score of ≥6, and the ability to undergo MRI. To limit variability that results from varying hematoma locations, only patients with basal ganglia hemorrhage were enrolled in this study. Exclusion criteria included patients with a GCS score of 3–5, planned surgical evacuation of a large hematoma (>40 ml), infection within 2 wk before admission, and admission at >24 h after onset. Also excluded were patients with hematoma expansion; secondary ICH; and concomitant use of antineoplastic, immunosuppressive, or immune-modulating therapies. We recruited volunteers as healthy controls who were matched by age, gender, body mass index, and other medical histories with patients with ICH as indicated in **Table 1**.

**TABLE 1.** Baseline characteristics of patients with ICH and healthy controls

Characteristic	Healthy controls	Patients with ICH	Patients SS < 37 ml	Patients SS > 37 ml	*P*
Participants (*n*)	20	39	20	19	
Age (yr)	59.1 ± 9.8	62.1 ± 11.8	63.3 ± 12.8	61.2 ± 9.0	n.s.^*a*^; n.s.^*b*^
Female [*n* (%)]	5 (25)	8 (21)	5 (25)	3 (16)	n.s.^*a*^; n.s.^*b*^
Body mass index	25.7 ± 3.5	26.4 ± 3.2	26.7 ± 3.1	25.2 ± 3.3	n.s.^*a*^; n.s.^*b*^
History of alcohol [*n* (%)]	10 (50)	13 (33)	5 (25)	8 (42)	n.s.^*a*^; n.s.^*b*^
Medical history [*n* (%)]					
Previous ICH	—	6 (15)	5 (25)	1 (5)	n.s.^*a*^
Ischemic stroke	—	7 (18)	4 (20)	3 (16)	n.s.^*a*^
Hypertension	2 (10)	35 (90)	19 (95)	16 (84)	n.s.^*a*^
Diabetes mellitus	3 (15)	6 (15)	3 (15)	3 (16)	n.s.^*a*^
Medication [*n* (%)]					
Warfarin anticoagulation	—	0 (0)	0 (0)	0 (0)	n.s.^*a*^
Antithrombotic treatment	—	0 (0)	0 (0)	1 (5)	n.s.^*a*^
Time to enrollment (h)	—	11.7 (8.5)	11.1 (9.1)	12.2 (8.2)	n.s.^*a*^
Clinical features					
Blood pressure (mmHg)	—				
Systolic	—	160 ± 20	160 ± 20	161 ± 21	n.s.^*a*^
Diastolic	—	97± 22	89 ± 16	101 ± 25	n.s.^*a*^
On admission	—				
GCS score	—	13.4 ± 1.6	13.5 ± 2.1	13.1 ± 1.4	n.s.^*a*^
NIHSS score	—	6.0 ± 5.1	5.7 ± 4.7	6.3 ± 4.1	n.s.^*a*^
Hematoma volume (ml)	—	10.9 ± 9.6	7.9 ± 6.5	14.1 ± 11.3	0.04^*a*^
Location of hematoma (lobar/deep^*c*^)	—	0/35	0/20	0/19	n.s.^*a*^
Intraventricular extension (*n)*	—	3	1	2	n.s.^*a*^

Data are shown as means ± sd except for items of percentage as identified. N.s., not significant; SS, splenic shrinkage. ^*a*^Patients with splenic shrinkage >37 ml *vs*. patients with SS <37 ml. ^*b*^Healthy controls *vs.* patients with ICH. ^*c*^Location designated as deep includes basal ganglia or thalamus, whereas the cerebellum and brain stem are excluded.

All eligible patients had a noncontrast head computed tomography scan upon admission (<24 h) and subsequent spleen and brain MRI at d 3 and 14 after ICH diagnosis. Peripheral blood samples were collected from patients with ICH during admission and at ∼7:00 AM on d 3 and 14. Blood samples were collected from healthy controls at 7:00 am after recruitment. Each spleen’s volume was measured on T2 images of the abdomen. Intravoxel incoherent motion (IVIM) diffusion-weighted imaging (DWI) (21) was performed to calculate the static tissue molecular diffusion (*D*, true diffusion coefficient, which reflected cell activity) and perfusion-related pseudodiffusion (D*, pseudodiffusion coefficient, reflecting the velocity of capillary blood). PHE was quantified by fluid attenuation inversion recovery (FLAIR) images of the brain; lymphocyte subsets of the blood were determined by flow cytometry. The study protocol was approved by the ethics committee of Tianjin Third Central Hospital and Tianjin Medical University General Hospital. Written informed consent was obtained from each participant or a legally acceptable surrogate before inclusion.

### MRI protocol

MRI scans were performed at our institutions with 3T Siemens Verio MRI Scanner (Siemens, Erlangen, Germany). MRI scans were characterized by the following parameters: FLAIR sequence of brain (repetition time/echo time, 6500/94 ms; 20 contiguous sections; 464 × 512 matrix; field of view, 24 cm; slice thickness, 6 mm); T2 half-Fourier acquisition single-shot turbo spin echo axial sequence of the spleen (repetition time/echo time, 2000/83 ms; 19 contiguous sections; 259 × 320 matrix; field of view, 38 cm; slice thickness, 6 mm); and IVIM-DWI of the spleen (spin-echo prepared echo-planar imaging scan in transverse orientation during free breathing with 9 *b* values, repetition time/echo time, 4500/68 ms; 19 contiguous sections; 80 × 128 matrix; field of view, 38 cm; slice thickness, 6 mm; *b* = 0, 50, 100, 150, 200, 400, 600, 800, and 1000).

Hematoma volume (Hv) and PHE volume (Ev) were measured on FLAIR. Relative PHE (rPHE) was defined as Ev/Hv. Hv and Ev were as outlined on the FLAIR slices and calculated for each slice from the measured area and corresponding slice thickness by using MIPAV software (National Institutes of Health, Bethesda, MD, USA). Spleen volumes were outlined on the T2 half-Fourier acquisition single-shot turbo spin echo slices of abdomen and calculated for each slice from the measured area and corresponding slice thickness by using MIPAV software.

The biexponential model was mathematically expressed as follows to analyze IVIM-DWI: S (*b*) = S_0_ (*f* × e^−^*^b^* ^×^ ^D*^ + (1−f) × e^−^*^b^* ^×^ *^D^*), where *f* is the unit of perfusion volume fraction, D* is the pseudodiffusion coefficient that reflects tissue capillary perfusion, and *D* is the true diffusion coefficient that reflects tissue cell activity. The diffusion quantification and analysis of IVIM were conducted by using MITK-Diffusion software (*http://www.mitk.org/*). A region of interest (ROI) analysis was performed to compute signal-intensity curves as a function of *b*. Splenic ROI was sampled at the middle of the spleen in a slice at the level of the hilus. Care was taken to avoid large vessels, blurred regions, and any focal lesions. The same ROI mask was propagated to images of all *b* values. Two independent radiologists who were blinded to the control analyzed all image data.

### Quantification of lymphocytes, neurotransmitters, and stress hormones in the circulation

Lymphocyte subset analyses were performed on fresh whole-blood samples from all human participants. Isolated mononuclear cells were immunostained with fluorochrome-conjugated Abs. The following Abs to human antigens were used: CD3-PerCP, CD4-FITC, CD8-PE, CD19-PE, CD56-APC, CD11b-PerCP, and CD11c-APC (BD Biosciences, Franklin Lakes, NJ, USA). Data were acquired by using a FACSCalibur (Becton Dickinson, San Jose, CA, USA) and analyzed with FlowJo software (Tree Star, Ashland, OR, USA).

Plasma levels of the neurotransmitters, acetylcholine and norepinephrine, and the glucocorticoid hormone, cortisol, were measured by ELISA (Abnova, Taipei, Taiwan) according to the manufacturer’s instructions. Optical densities were measured at 450 and 570 nm.

### Experimental ICH study

Adult male C57BL/6 mice (10–12 wk old) were used for animal experiments. For the collagenase model, ICH was induced by intracerebral injection of 0.0375 U collagenase (type IV-S; Sigma-Aldrich, St. Louis, MO, USA) at the site of the right basal ganglia (4). To induce ICH of varied volumes in mice, graduated doses of collagenase were used: 0.01, 0.035, and 0.06 U. For the autologous blood injection model, mice were injected with 30 μl of autologous blood in the right basal ganglia site, as previously described (22). Spleens of mice with ICH were weighed at the indicated time points immediately after euthanasia. For pathologic staining, procedures were completed as previously described (4, 23). In brief, mice of the 0.0375 U collagenase injection model were humanely killed at d 3 after ICH induction, and spleens were quickly removed and fixed in 4% paraformaldehyde overnight followed by dehydration with 30% sucrose until the tissue precipitated to the bottom. Incubation in optimal cutting temperature compound followed. Then, 8-μm-thick cross-sections were cut on a cryostat (LM3050S; Leica Microsystems, Wetzlar, Germany) and were mounted on poly-l-lysine–coated slides. Each tissue section was stained with hematoxylin and eosin to measure the white and red pulp areas of the spleen. Pictures were acquired with a microscope (Nikon, Tokyo‎, Japan) and analyzed by using Image Pro Plus (Media Cybernetics, Rockville, MD, USA). For neuropathway blocking experiments in mice, the following antagonist regimens were used: RU486 (Sigma-Aldrich) was dissolved in ethanol/sesame oil solution (1:10, 6 mg/ml) and intraperitoneally injected at 30 mg/kg body weight at 24 and 5 h and immediately before ICH induction (24). Propranolol (Sigma-Aldrich) was dissolved in saline at 6 mg/ml and intraperitoneally injected at 30 mg/kg immediately after model induction and repeated at d 1 and 2. Dihydro-β-erythroidine (DHβE) was dissolved in saline at 0.1 and 1 mg/kg and was injected i.p. immediately after ICH induction. Spleen weight was measured at d 3 after model induction. Animal experiments were approved by Animal Care and Use Committees of the Barrow St. Joseph or Tianjin Neurologic Institute.

### Statistics

Statistical analysis was performed by using SPSS software for Windows (v.17.0; SPSS, Chicago, IL, USA). Results are expressed as means ± sd for continuous variables and as probability for categorical variables. Between 2 groups, nonparametric data were compared by using the Mann-Whitney test, and parametric data were compared with Student’s *t* test. In addition, 1-way ANOVA was performed to compare parametric data among more than 3 groups with *post hoc* tests, and 2-way ANOVA was used to compare repeated measurement-to-data between 2 groups. A χ^2^ test was used to analyze differences in categorical variables. Spearman correlation analysis was conducted to assess factors that may contribute to the occurrence of spleen and lymphocyte alteration. A value of *P* < 0.05 was considered significant.

## RESULTS

### Characteristics of participants

A total of 39 patients with ICH were enrolled in this study between August 2015 and May 2016. There were no fatalities, no missing follow-ups, and no dropouts. Mean time from onset of ICH to enrollment was 11.7 ± 8.5 h. Mean age of patients enrolled was 62.1 ± 11.8 yr, and the male:female ratio was 31:8. The 20 healthy controls had a mean age of 59.1 ± 9.8 yr and a male:female ratio of 15:5. There was no significant difference for age, gender, and body mass index between patients with ICH and healthy controls. The demographic, clinical, and radiologic characteristics of study participants are listed in Table 1.

### Spleen shrinkage after ICH

**Figure 1.**
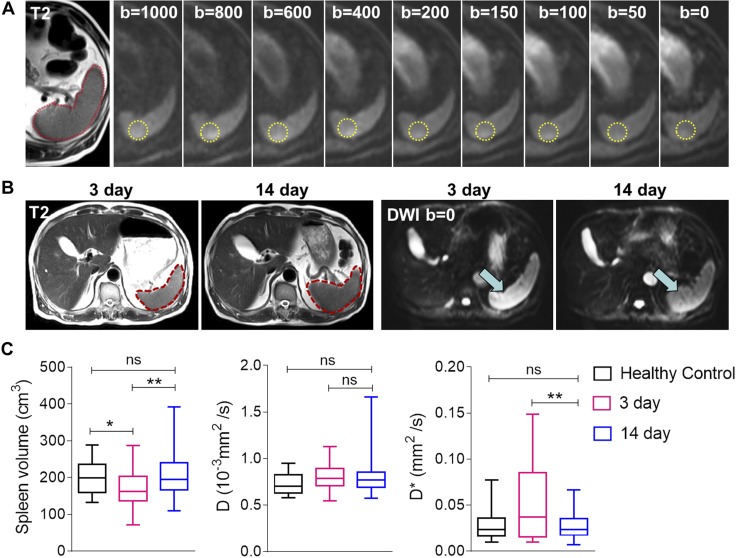
Spleen shrinkage and increased capillary perfusion after ICH onset. *A*) Representative images of abdominal MRI with T2-weighted images and DWI at 9 consecutive *b* values from 0 to 1000. Spleen volumes were calculated from T2 images. Red outlines show the spleen, and yellow circles show the ROI for measuring signal intensity of the spleen from DWI images at different *b* values—signal density decayed with increasing *b* values. IVIM-DWI parameters, *D* and D*, were derived from these multiple DWIs. *B*) Representative abdominal MRIs from a patient with ICH at d 3 and 14 after ICH show an enlarged spleen at d 14 compared with d 3 (spleen is outlined in red; right). DWI images at *b* = 0 of the same patient show that the signal density of the spleen at d 3 was higher than at d 14, which indicates increased perfusion at d 3 post-ICH (left). *C*) Quantitative analysis shows dynamic changes of spleen volume after ICH onset. *D* and D* values of spleen are compared; the horizontal line inside each box indicates the median. The top and bottom of the box indicate the interquartile range, and error bars indicate the 5th and 95th percentiles. Comparisons were performed by using the Mann-Whitney test. Ns, not significant. *P < 0.05, **P < 0.01.

The spleen is a major secondary lymphoid organ that serves as a peripheral reservoir of innate and adaptive immune cells where immune cells are uniquely organized (25). The spleen swiftly responds to infection but also to severe body injury, including stroke and traumatic brain injury. The measurement of spleen volume in patients with ICH at d 3 after stroke showed smaller spleens than at d 14 in the same patient, and at d 14 spleen volumes of patients with ICH were comparable in size to those from healthy controls (**Fig. 1*B, C***). As a result of kinetics, these data are not in conflict with those from a previous study that reported that spleen volumes of patients with ICH at d 5 after ischemic stroke were similar to those at d 90—when the spleen volume was regarded as normal (26). In calculating the extent of spleen shrinkage as the difference between spleen volumes at d 3 *vs.* 14, we found an average spleen shrinkage volume of 37 cm^3^ (*i.e.*, a 17.2% decrease in average spleen size at d 3 after ICH induction).

The IVIM-DWI approach has been used in reproducible studies on liver and kidney (21, 27) that modeled the diffusion-attenuated MRI signal as a sum of static tissue molecular diffusion and perfusion-related pseudodiffusion (21). The true diffusion coefficient, *D*, represents the static tissue molecular diffusion, which indirectly reflects the cell activity of the tissue, whereas the pseudodiffusion coefficient, D*, reflects the perfusion at the capillary level. Nine masks were drawn by hand in the spleen as DWI for all *b* values with good quality and reproducibility (Fig. 1*A*). D* and *D* values were not significantly different between healthy controls and patients at d 14 after ICH induction; however, patients’ D* values were higher at d 3 compared with d 14 (*P* < 0.001), whereas the *D* value was stable (Fig. 1*C*). Thus, despite a smaller spleen size, capillary blood perfusion of the spleen increased by d 3 after ICH induction.

### Spleen shrinkage can be predicted by hematoma size at the patient’s admission, and correlates with PHE and ICH outcomes

**Figure 2.**
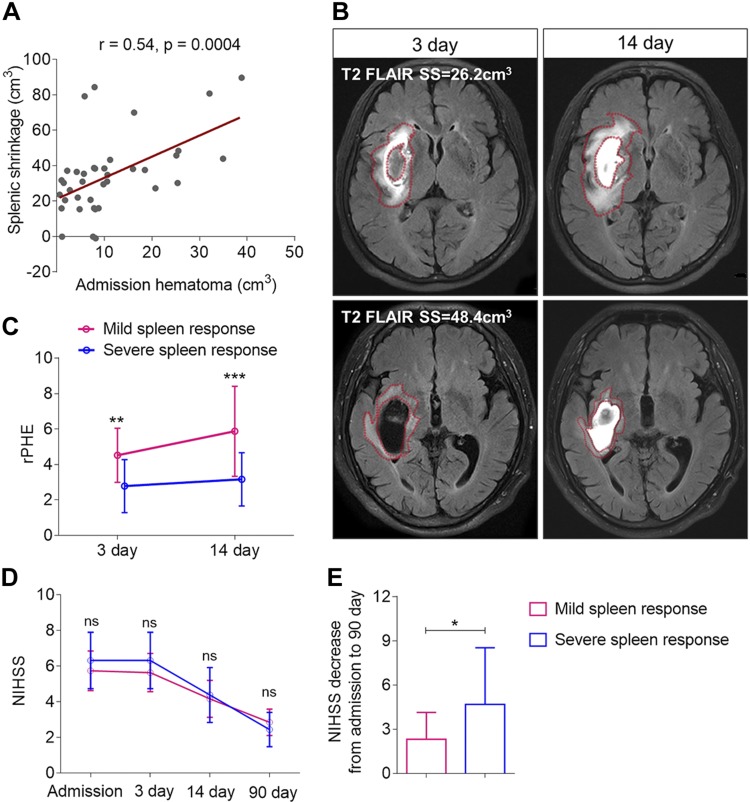
Spleen shrinkage is associated with hematoma size at admission and with progression of PHE. *A*) Association between the extent of spleen shrinkage and hematoma volume at admission. Spleen shrinkage equals spleen volume at d 14 minus spleen volume at d 3 on MRI. All 39 patients with ICH were included. Spearman *r* and *P* values show correlations. Patients were subdivided into 2 groups according to the average extent of spleen shrinkage. Spleen shrinkage of >37 cm^3^ was considered to be severe, and spleen shrinkage of <37 cm^3^ to be mild. *B*) Representative brain MRI scans at T2 FLAIR of 2 patients with spleen shrinkage volumes of 26.2 or 48.4 cm^3^, respectively, showing PHE as red outlines. *C*) rPHE of patients measured by MRI at T2 FLAIR sequence at d 3 and 14 after ICH onset. rPHE was calculated as PHE divided by hematoma. Two-way repeated measurements ANOVA was used for comparison. Data are shown as means ± sd. *D*) NIHSS score changes at the indicated time points are shown as means ± sd (2-way ANOVA). *E*) Comparison of NIHSS decrease from admission to 90 d. ns, not significant; SS, spleen shrinkage. *P < 0.05, **P < 0.01, ***P < 0.001 (Student’s *t* test).

Day-to-day variability in healthy human spleen size has been measured *via* abdominal ultrasound (28). The maximum reported change in median spleen volume in healthy individuals between any 2 consecutive days has been suggested to be 10.5 cm^3^, and median changes over the course of 5 d has been indicated to be 1.5 cm^3^. Here, we found that the average reduction of spleen volume in patients with ICH was 37 cm^3^, which is larger than the normal daily variability of 10.5 cm^3^. To explore clinically and immunologically the finding of spleen shrinkage after ICH induction, we arbitrarily divided patients with ICH into 2 groups: one with severe spleen shrinkage (>37 cm^3^; *n* = 19), and one with lesser spleen shrinkage (<37 cm^3^; *n* = 20). Demographics, risk factors, and clinical features (Table 1) were all similar between patients with severe or moderate spleen shrinkage; however, differences were noted for hematoma volume at admission. In particular, mean hemorrhage volume at admission for patients with severe spleen shrinkage was greater than that for patients with lesser shrinkage (14.1 ± 11.3 *vs.* 7.9 ± 6.5 cm^3^; *P* < 0.05). Moreover, hemorrhage volume at admission (<24 h) was associated with spleen shrinkage at d 3 after ICH induction (*r* = 0.54; *P* < 0.01; **Fig. 2*A***).

Although hematomas generally enlarge within 3 h after ICH onset and become stable within 24 h, PHE increases continuously for up to 2 wk (1). In addition, conspicuous edema in relation to the volume of hemorrhage is correlated with worse clinical outcomes (29). Because spleen shrinkage is associated with hematoma volume at admission for patients with ICH, we assessed the possible correlations between spleen shrinkage and PHE. Figure 2*B* shows that PHE in patients with lesser spleen shrinkage expanded rapidly in the first 3 d after ICH onset, and this trend continued for up to 14 d. In contrast, patients with severe spleen shrinkage had milder and slower expansion of edema in the 3 d post-ICH period, and those levels remained constant during the next 11 d. Incidentally, rPHE was smaller in patients with ICH with severe spleen shrinkage compared with those with lesser spleen shrinkage—at both d 3 and 14 after ICH induction (2.8 ± 1.5 *vs.* 4.5 ± 1.5; *P* < 0.001; and 3.2 ± 1.5 *vs.* 5.8 ± 2.5; *P* < 0.01; Fig. 2*C*).

Associations between spleen shrinkage and ICH outcomes (Fig. 2*D*) indicate that, although the U.S. National Institutes of Health Stroke Scale (NIHSS) score from admission to 90 d did not differ significantly between patients with severe or moderate spleen shrinkage, the decrease in score from admission to 90 d was more prominent in those patients in whom spleen volume decreased >37 cm^3^ (*P* < 0.05; Fig. 2*E*). This indicates that patients with severe spleen shrinkage might have a relatively better recovery from neurologic deficits; however, the modified Rankin scale (mRS) of 0–1 at 90 d between the 2 groups did not find any differences that reached statistical significance (data not shown).

As hematoma size upon admission can predict the extent of spleen shrinkage in patients with ICH, and severe spleen shrinkage is associated with milder progression of rPHE, these sequential events might hasten neurologic recovery in patients with ICH.

### T- and NK-cell deficiency after ICH is associated with increased infection risk and poorer clinical outcomes

**Figure 3.**
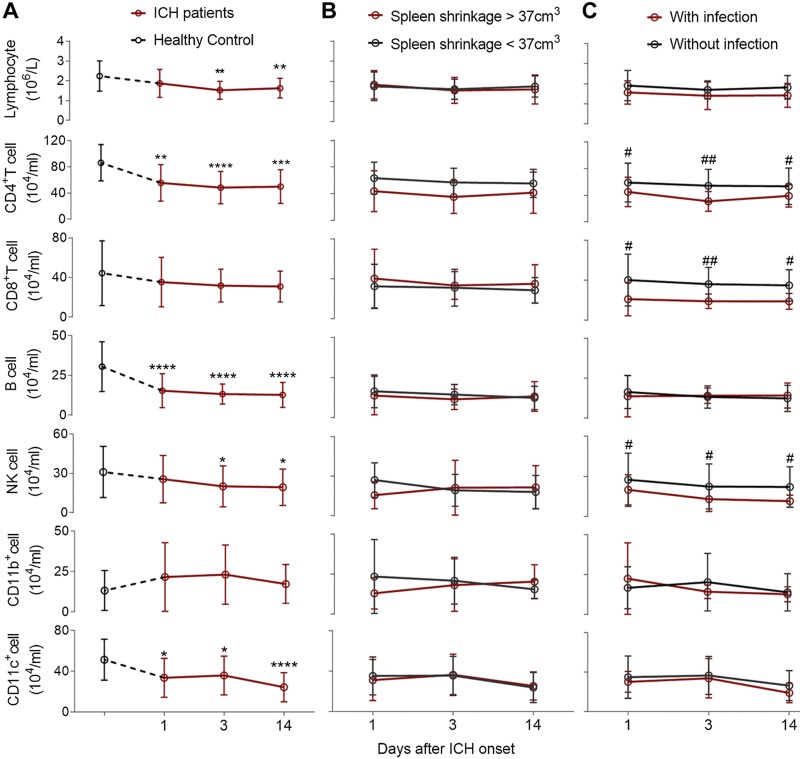
Persistence of lymphopenia after ICH onset and association between lymphocyte deficiency and increased susceptibility to infection. *A*) Lymphocyte and monocyte subset counts in the peripheral blood of healthy controls (black) and patients with ICH (red) at admission and 3 and 14 d after ICH. Lymphocyte counts were inferred from routine blood tests. All other cell subsets were detected *via* flow cytometry, following gating strategies: CD3^+^CD4^+^ for CD4^+^ T cells; CD3^+^CD8^+^ for CD8^+^ T cells; CD3^−^CD19^+^ for B cells; CD3^−^CD56^+^ for NK cells; CD11b^+^ for monocytes; and CD11c^+^ for dendritic cells. Patients with ICH were subdivided into groups according to the extent of spleen shrinkage and the presence or absence of infection during hospitalization after ICH. *B*) Comparison of immune cell subset counts in the blood in relation to spleen shrinkage. *C*) Association between infection and immune cell subset counts. Data are shown as means ± sd. Comparisons were performed by using Student’s *t* test. *P < 0.05, **P < 0.01, ***P < 0.001, ****P < 0.0001 (ICH patients *vs.* healthy controls); ^#^*P* < 0.05, ^##^*P* < 0.01 (patients with infection *vs*. patients without infection).

**Figure 3*A*** shows that the number of lymphocytes and CD4^+^T, NK, B, and CD11C^+^ cells decreased after the onset of ICH; thus, peripheral lymphopenia after ICH impacts both the innate and adaptive arms of the immune system. With the exception of 1 subset, cell counts reached a nadir at d 3 that remained until d 14. Although CD8^+^T cells also displayed a negative trend after ICH onset, differences were not statistically significant. When patients with ICH with spleen shrinkage of >37 cm^3^ were compared with patients with spleen shrinkage of <37 cm^3^, total lymphocyte counts and/or subset frequency did not display differences (Fig. 3*B*).

To relate the findings to the clinic, we assessed the incidence of infection in 39 patients with ICH. Twelve patients (31%) suffered infection during hospitalization, including 1 with a urinary tract infection, 1 who had both a respiratory infection and urinary tract infection, and the remaining 10 patients all with respiratory infections. The comparison of admission characteristics and outcomes in patients with and without infection demonstrated that patients with and without infection had similar age, GCS scores at admission, spleen volume at d 3 and 14 post-ICH, and similar spleen shrinkage (**Table 2**). At admission, the hematoma size of patients who suffered infection seemed to be larger than that in patients without infection; however, such differences did not reach statistical significance (13.5 ± 9.5 *vs*. 9.9 ± 9.6 cm^3^; *P* > 0.05). Of importance, more severe neurologic deficit was evident in patients with infection, and this, in turn, impacted the 3-mo outcome: 70% of patients without infection had better outcomes compared with 8% of infected patients (*P* < 0.0001). In addition, CD4^+^T, CD8^+^T, and NK cell counts were significantly lower from d 1 to 14 in those patients with infection (Fig. 3*C*). Our results are in agreement with a prospective study of 2014 patients with ICH that found that lymphopenia was common in these patients and was associated with infectious complication (30).

**TABLE 2.** Characteristics of patients with ICH with and without infection

Characteristic	Without infection	With infection	*P*
Participants (*n*)	27	12	
Age (yr)	60.9 ± 11.4	65.1 ± 10.4	n.s.
Female [*n* (%)]	5 (19)	3 (25)	n.s.
Clinical features			
Hematoma volume at admission (cm^3^)	9.9 ± 9.6	13.5 ± 9.5	n.s.
GCS score at admission	14.9 ± 0.6	13.3 ± 2.4	n.s.
NIHSS score			
Admission	4.8 ± 4.4	11.7 ± 4.9	0.0003
3 d	4.7 ± 4.2	12.1 ± 4.7	0.0001
14 d	3.1 ± 3.8	9.3 ± 5.2	0.0003
90 d	2.1 ± 2.9	5.9 ± 3.4	0.0008
mRS score			
Admission	4.4 ± 0.5	2.4 ± 1.4	0.0001
3 d	4.4 ± 0.5	2.4 ± 1.4	0.0001
14 d	1.8 ± 1.3	3.8 ± 0.7	<0.0001
90 d	1.0 ± 1.3	2.8 ± 0.8	0.0001
mRS 0–1 on 90 d [*n* (%)]	19 (70)	1 (8)	0.0006
Spleen volume (cm^3^)			
3 d	175.9 ± 68.4	164.6 ± 37.9	n.s.
14 d	223.7 ± 100.2	200.7 ± 44.6	n.s.
Spleen shrinkage volume	47.8 ± 44.2	36.1 ± 16.5	n.s.
rPHE			
3 d	4 ± 1.6	2.9 ± 1.5	n.s.
14 d	4.9 ± 2.4	4.5 ± 2.8	n.s.

Means ± sd throughout except for those specifically identified; n.s., not significant.

### Synergy of sympathetic and hypothalamus-pituitary-adrenal axis mediates spleen responsiveness after ICH onset

**Figure 4.**
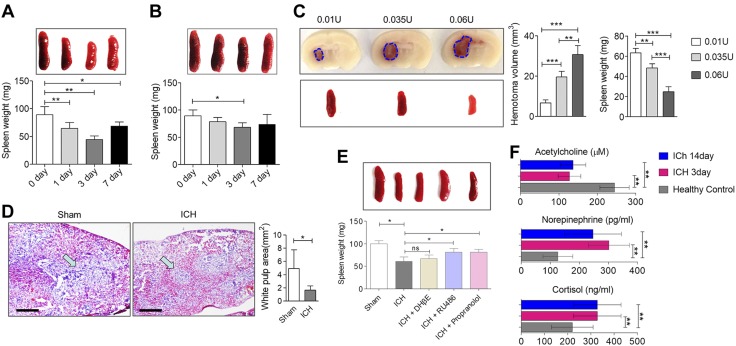
Spleen shrinkage after ICH is a result of synergism between the adrenergic and HPA axis. *A*, *B*) Spleen weight changes at indicated time points in mice with ICH induced by 0.0375 U collagenase (*A*) or 30 μl autologous blood (*B*) injected into the right basal ganglia of male adult C57BL/6 mice. Means ± sd and Student’s *t* test (*n* = 6 for each time point). *C*) Representative images of brain hematoma and spleen of mice that were injected with different doses of collagenase (left). Hematoma volume and spleen weight of mice that were injected with different doses of collagenase (right; means ± sd, 1-way ANOVA, *n* = 6 per group). *D*) Representative hematoxylin and eosin staining of spleen from mice with ICH induced by intracerebral injection of 0.0375 U collagenase (left). Sham-treated controls were injected with PBS. Scale bars, 200 μm. Comparison of splenic white pulp area (right; means ± sd, Student’s *t* test, *n* = 9 for each group). *E*) Representative images of spleen and spleen weight of collagenase-injected mice with ICH that were treated with the indicated blockers. Spleen weights were measured at d 3 (means ± sd, 1-way ANOVA, *n* = 6 in each group). *F*) Plasma concentration of neurotransmitters and glucocorticoid hormones detected by ELISA at d 3 and 14 after ICH (means ± sd, 1-way ANOVA). ns, not significant; RU486, mifepristone. *P < 0.05, **P < 0.01, ***P < 0.001.

We used 2 experimental models of ICH. In the first model, after collagenase injection of mice with ICH, spleen weight significantly decreased at d 1, 3, and 7 (**Fig. 4*A***). In the second model of ICH, after injection of autologous blood, mice developed significant loss of spleen weight at d 3 and partially recovered by d 7 (Fig. 4*B*). With the administration of increasingly larger doses of collagenase to induce larger hematomas, we observed that the larger the dose of collagenase was associated with larger hematomas, as expected, as well as with increased spleen shrinkage (Fig. 4*C*). These results confirm what was observed in patients with ICH, namely, that hematoma size is a predictor of spleen shrinkage. In addition, we analyzed the lymphocyte change in the blood and spleen of mice with ICH. Similar to findings for patients with ICH, the peripheral lymphocyte subset counts of mice with ICH were significantly lower than sham-treated control at 1 d after model induction, which was sustained until 7 d. In the spleen, the same populations of cells were also decreased as early as 1 d after ICH induction in mice; however, compared with circulation, the cell number of these cells in the spleen recovered at 7 d after ICH onset (Supplemental Fig. 1).

MRI data from splenic scans implied that blood perfusion of spleen capillaries increased 3 d after ICH onset, whereas true tissue diffusion was stable. Hematoxylin and eosin staining of spleens demonstrated that the red pulp remained segregated from the white pulp in sham-treated mice (Fig. 4*D*), but in mice with ICH the blood brimmed to a point that large numbers of erythrocytes could be observed inside vessels and the white pulp was obliterated (*P* < 0.05). As severe brain injuries have been described to activate the hypothalamic-pituitary-adrenal (HPA) axis and the sympathetic nervous system with a subsequent release of adrenal steroid hormones and catecholamines that are immunosuppressive (24), we investigated the neural pathways in the spleen after ICH onset. We also investigated cholinergic innervation as it is also involved in the neurogenic modulation of immunity (31). We used the β-adrenergic receptor blocker, propranolol, for adrenergic blockade, RU486 for glucocorticoid receptor blockade, and α4β2 cholinergic blocker, DHβE, for cholinergic blockade. Results demonstrate that DHβE did not influence spleen responses, whereas propranolol and RU486 partially inhibited spleen shrinkage induced by ICH onset (Fig. 4*E*). In addition, plasma acetylcholine levels were lower in patients with ICH compared with healthy controls, remaining stable from d 3 to 14, whereas serum norepinephrine and cortisol were higher in patients with ICH at d 3 and 14 (Fig. 4*F*). The determination of plasma levels of acetylcholine, norepinephrine, and cortisol in mice with ICH also suggested the prolonged activation of the HPA axis and adrenergic pathway (Supplemental Fig. 2). These results suggest a synergy between sympathetic innervation and the HPA axis in ICH-induced immune effects.

## DISCUSSION

Here, we document quantitative and temporal changes in the peripheral immune system after ICH. A reduction of spleen volume occurred with a concurrent increase in capillary perfusion—the timing of this alteration was between d 3 and 14 after stroke. In addition, during the 2-wk period after stroke, patients with ICH were lymphopenic and these events could be recapitulated in mouse models of ICH. Reduction of T and NK cells was associated with increased susceptibility to infection, which was associated with worsened functional outcomes. Finally, the neurogenic pathways that governed those events included a synergistic activation of the sympathetic nervous system and the HPA axis.

Spleen size has been measured after ischemic or hemorrhagic stroke *via* ultrasound scan (28, 32), with measurements of width, length, and thickness. Here, we more accurately assessed spleen volume by using MRI after ICH, together with IVIM-DWI, to monitor changes in tissue diffusion and alterations of splenic capillary perfusion (27, 33), and observed an initial reduction in the spleen volume of patients with ICH that was evident at d 3 and that recovered to normal size by d 14. The finding of no significant change of splenocyte activity—according the *D* value of IVIM-DWI—and the higher capillary perfusion—reflected by the D* value of IVIM-DWI—in patients with ICH at d 3 indicate an increase of the spleen’s blood exchange at d 3 when the spleen volume was reduced. ICH could induce an egress of spleen cells from the white pulp, as supported, in part, by the finding of a loss of splenic white pulp after experimental ICH induction.

We also found that in patients with ICH, plasma norepinephrine and cortisol were higher compared with controls until 14 d after ICH, and noradrenergic and/or HPA axis blockade inhibited, in part, spleen shrinkage after ICH. This suggests a persistent activation of the sympathetic nervous system and the HPA axis after ICH, and identifies pharmacologic targets of immune modulation in ICH. Conversely, it seems that spleen volume and lymphopenia were impacted differently by ICH. Lymphopenia was present at patient hospital admission and persisted throughout the 14-d observation, whereas the decreased spleen volumes returned to normal by d 14. In this context, the deficiency of T and NK cells was associated with an increased susceptibility to infection and worsened clinical outcomes. Almost one third of patients with ICH developed infectious complications during hospitalization, which is consistent with a study of approximately 800 patients with ICH (19), and those who suffered infections developed more severe neurologic deficit—assessed by NIHSS—and had worse 3-mo functional outcomes. However, spleen shrinkage was associated with a relatively milder progression of PHE and a relatively better recovery from neurologic deficit. Animal experiments suggested that spleen response post-ICH might participate in brain inflammation and worsen ICH outcome, whereas splenectomy can inhibit brain edema and improve neurologic deficit (34). Therefore, we speculate that spleen shrinkage post-ICH, as observed in this study, might serve as a means of sparing the brain from destruction by overwhelming inflammation.

In the presence of a neurologic insult, such as ICH, the brain allows large numbers of peripheral immune cells to infiltrate its parenchyma. Depending on the timing, those infiltrates can either damage neural structures or promote tissue repair (3, 35, 36). Reciprocally, the brain can also shape peripheral immune responses. A prevailing idea is that brain-derived neurogenic innervations can modulate systemic immunity (24, 37, 38). Brain injury–induced activation of neurogenic pathways includes the HPA axis, sympathetic innervation, and parasympathetic innervation, which may cooperate to influence the magnitude of immune response (24, 38). In support of this notion, we found that ICH-induced activation of the HPA axis and sympathetic nervous system contributes to immunosuppression in the periphery. This immune suppression might inhibit the overwhelming of brain inflammation, but with the cost of increased risk of infection because of the disruption of immune defense. This finding has clinical implications for the management of patients with ICH. In a 2-arm proof-of-concept clinical study in patients with ICH within 72 h of stroke, the immune modulator, fingolimod, which inhibits the egress of lymphocytes from lymph nodes, was found to improve short- and long-term neurologic functions by inhibiting PHE progression (10). The current study adds that immune modulators, such as like fingolimod, should be administered early in ICH, as long-term use may exacerbate immune suppression. In addition, considering the high prevalence and morbidity of infection in patients with ICH and the unclear benefits of antibiotics in those patients (19, 20), our finding that pharmacologic modulation of neurogenic innervations can preserve spleen size and cellular immunity after ICH may provide an opportunity to improve immune defenses against post-ICH infection—a possible approach for the prevention of post-ICH infection. Because our results are preliminary as a result of the small sample size, additional studies are encouraged to investigate the precise mechanisms of immunosuppression after ICH and the benefit of neuromodulation to improve immune defense and clinical outcomes.

## Abbreviations

DHβE: dihydro-β-erythroidine
DWI: diffusion-weighted imaging
Ev: perihematomal edema volume
FLAIR: fluid attenuation inversion recovery
GCS: Glasgow coma scale
HPA: hypothalamus-pituitary-adrenal
Hv: hematoma volume
ICH: intracerebral hemorrhage
IVIM: intravoxel incoherent motion
mRS: modified Rankin Scale
NIHSS: U.S. National Institutes of Health Stroke Scale
PHE: perihematomal edema
rPHE: relative perihematomal edema
ROI: region of interest
